# Development of an Improved Rapidly Exploring Random Trees Algorithm for Static Obstacle Avoidance in Autonomous Vehicles

**DOI:** 10.3390/s21062244

**Published:** 2021-03-23

**Authors:** S. M. Yang, Y. A. Lin

**Affiliations:** Department of Aeronautics and Astronautics, National Cheng Kung University, Tainan City 70101, Taiwan; andrew093200@gmail.com

**Keywords:** autonomous vehicle obstacle avoidance, path planning, Rapidly Exploring Random Trees

## Abstract

Safe path planning for obstacle avoidance in autonomous vehicles has been developed. Based on the Rapidly Exploring Random Trees (RRT) algorithm, an improved algorithm integrating path pruning, smoothing, and optimization with geometric collision detection is shown to improve planning efficiency. Path pruning, a prerequisite to path smoothing, is performed to remove the redundant points generated by the random trees for a new path, without colliding with the obstacles. Path smoothing is performed to modify the path so that it becomes continuously differentiable with curvature implementable by the vehicle. Optimization is performed to select a “near”-optimal path of the shortest distance among the feasible paths for motion efficiency. In the experimental verification, both a pure pursuit steering controller and a proportional–integral speed controller are applied to keep an autonomous vehicle tracking the planned path predicted by the improved RRT algorithm. It is shown that the vehicle can successfully track the path efficiently and reach the destination safely, with an average tracking control deviation of 5.2% of the vehicle width. The path planning is also applied to lane changes, and the average deviation from the lane during and after lane changes remains within 8.3% of the vehicle width.

## 1. Introduction

According to the World Health Organization, there are 1.35 million fatalities due to traffic accidents each year [1]. With the advances in mobile communication technology, advanced driver assistance systems and intelligent transportation systems are under development to reduce traffic accidents caused by driver negligence. The concept of the Internet of Vehicles allows vehicles to drive autonomously, reduces the operator’s burden, and improves driver safety. This is making autonomous vehicles possible through high data transmission efficiency and low transmission latency [2]. To ensure safety, an autonomous vehicle must have the ability to identify and avoid obstacles.

Safe path planning is key in autonomous vehicles. An autonomous vehicle has a perception layer to detect its location, a planning layer to predict the safe path/route, and a control layer to maneuver the vehicle’s direction and speed [3,4,5]. Sensors such as receivers of the Global Navigation Satellite System (GNSS), inertial measurement units (IMUs), LiDAR, cameras, and radars were all installed to investigate vehicles in an obstacle-free environment [6,7]. Simultaneous location and mapping by normal distribution transform was proposed for vehicles in deeply urbanized cities [8]. The simulation of the sensor uncertainties influencing the path planning was conducted in [9]. The methods of path planning can be summarized into four categories: the graph search method, the sampling method, the interpolating method, and the numerical optimization method. Among them, numerical optimization using deep learning neural networks (DNN) has been the recent focus [10]. The Markov random field model was applied to path planning [11], but as in any numerical optimization, the computational load is often too heavy for real-time applications at present. Recent studies proposed the application of potential fields to path planning optimization [12] with fuzzy logic [13] or with a Kalman-filter-like observer [14]. An adaptive potential field was recently developed for autonomous vehicles in complex driving scenarios such as emergency braking and accelerating [15]. Again, most of the above were limited to numerical simulation without any tracking/speed control and computation loading concerns. Hybrid path planning for optimization was also reviewed [16]. Hybrid path planning that employed fuzzy logic in decision-making was applied to generate virtual waypoints for path optimization [17], and another hybrid path planning approach combining a potential field with a sigmoid curve was proposed to improve vehicle stability and ride comfortability [18]; however, they remain limited to numerical simulation. Furthermore, some have proposed the use of the convex model to decompose the operating space into several regions [19], or the use of potential fields to improve path planning efficiency [20]. 

By comparison, the Rapidly Exploring Random Trees (RRT) algorithm is a feasible, relatively fast, and compatible solution in a searching space which avoids colliding with obstacles [21]. It has mainly been applied to robot maneuvering [22,23,24,25,26] and modified to adapt to trajectory curvature constraint [27], collision detection [28], quicker planning by the triangular inequality method [29], and to consider the bias goal factor [30]; however, the above were all carried out in numerical simulation only. Recent advances in the RRT algorithm have been extending to path planning of autonomous vehicles by numerical simulation [31,32]. The hardware constraints and the computation speed in tracking the planned path have been seldom investigated. In addition, the path obtained by the RRT algorithm has many unnecessary turning points that make path planning inefficient [19,32]. Path pruning is therefore needed to reduce the number of turning points. However, the path after pruning remains infeasible for vehicle tracking because it is not continuously differentiable. Path smoothing is also needed. Furthermore, the path predicted by the RRT algorithm may not be efficient (in terms of path/track length) because of the algorithm’s random nature. An improved algorithm is necessary to construct a safe, smooth path with optimal distance from start to destination without colliding with obstacles. This work is organized as follows. Section 2 briefly introduces the RRT algorithm, highlighting the need for an improved algorithm with pruning, smoothing, and optimization. Section 3 proposes the pruning process in reducing the number of waypoints from a random search. Section 4 employs the Fibonacci number in defining the waypoints for a Bézier curve to carry out trajectory smoothing and optimization. Finally, Section 5 reports the experiments conducted on an autonomous vehicle to verify the effectiveness of the improved RRT algorithm through tracking control, lane change, and lane keeping.

## 2. Rapidly Exploring Random Trees (RRT) Algorithm

**Figure 1:**
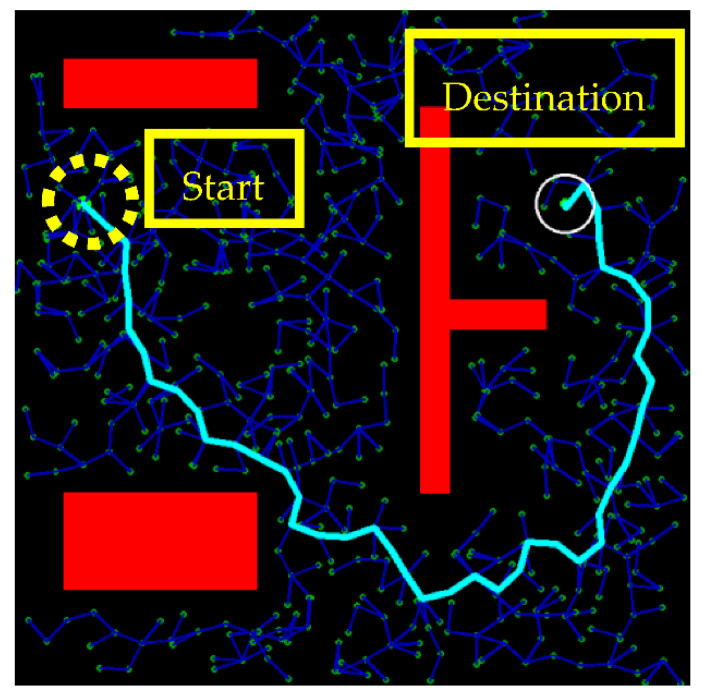
Illustration of the RRT algorithm with the tree expanding from the start $x_{start}$ on the upper left to the destination $x_{dest}$ on the upper right. The thin lines and points are the roots of the searching tree and the thick line is the path predicted by the algorithm. Note that the predicted path has many redundant points, unnecessary turns, and small curvatures infeasible for vehicle operation.

The RRT algorithm is used to construct a path from the start $x_{start}$ to the destination $x_{dest}$ in a metric space X by searching the free space $X_{free}$, $X_{free}\subset X$, away from the obstacle $X_{obs}$, $X_{obs}\subset X$. Consider that $x_{start}$ and $x_{dest}$ are both points in two-dimensional space. The RRT algorithm starts from $x_{start}\;$ and randomly samples a point $x_{rand}$ to find the nearest neighboring point $x_{near}$ to construct a tree. The definition of “nearest” is the shortest distance in Euclidean distance, such that $x_{near}$ is in the direction toward $x_{rand}$ at a distance for the new point, also called the node, $x_{new}$. If $x_{new}\subset X_{free}$, the tree expansion continues, and the algorithm connects $x_{near}$ to $x_{new}$ and checks if the connection collides with any obstacle $X_{obs}$. If it does, the algorithm restarts; otherwise, $x_{new}$ is added to the tree as a new point, and the search repeats until the tree reaches the destination or the number of iterations of expanding the tree reaches the limit. Figure 1 illustrates the RRT algorithm of expanding the searching tree so as to plan a safe path from the start to the destination.

Due to the random nature of the algorithm, there were many redundant turning points (nodes) in the path, as shown in Figure 1, and this made the path planning inefficient in reaching the destination. These redundant points were also the reason that the planned path was not continuously differentiable and hence infeasible for autonomous vehicle operation. Moreover, there was certainly more than one feasible path to the destination, but the random nature also made the search for the “optimal” path inefficient. Path smoothing and optimization are therefore needed.

## 3. Improved RRT Algorithm with Pruning

**Figure 2:**
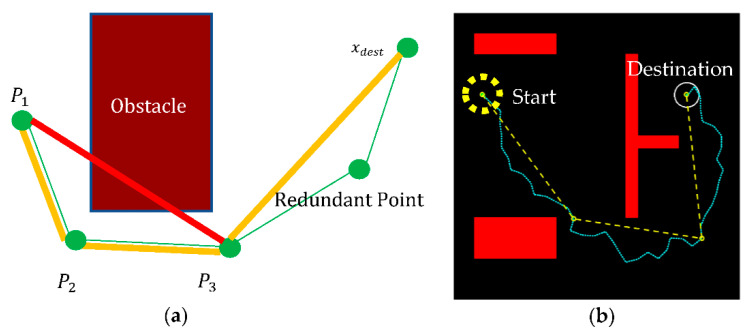
(**a**) Illustration of the pruning process by taking three consecutive path points $P_{1}$, $P_{2},$ and $P_{3}$ to check if the new connection $P_{1}$ to $P_{3}$ is safe. If so, $P_{2}$ is redundant; if not, $P_{2}$ remains the path point. (**b**) The path after the pruning process (dash line) compared with that of the RRT algorithm (dot line).

The path obtained by the RRT algorithm was plagued by the randomly generated nodes that can result in a poor connectivity path. Furthermore, the path was often not continuously differentiable and was thus infeasible for vehicle implementation. Pruning is a prerequisite for smoothing. The pruning process shown in Figure 2 was conducted to remove the redundant points in the RRT algorithm for a new path without colliding with obstacles. The process started from three consecutive path points, $P_{1}$, $P_{2},$ and $P_{3},$ as illustrated in Figure 2a. If there was no collision, $P_{1}$ and $P_{3}$ were directly connected as a new path and $P_{2}$ was redundant. Conversely, if a collision was found between $P_{1}$ and $P_{3}$, then $P_{2}$ was retained as the path point. The pruning process can remove redundant path points and obtain a more efficient path to the destination, as illustrated in Figure 2b.

**Figure 3:**
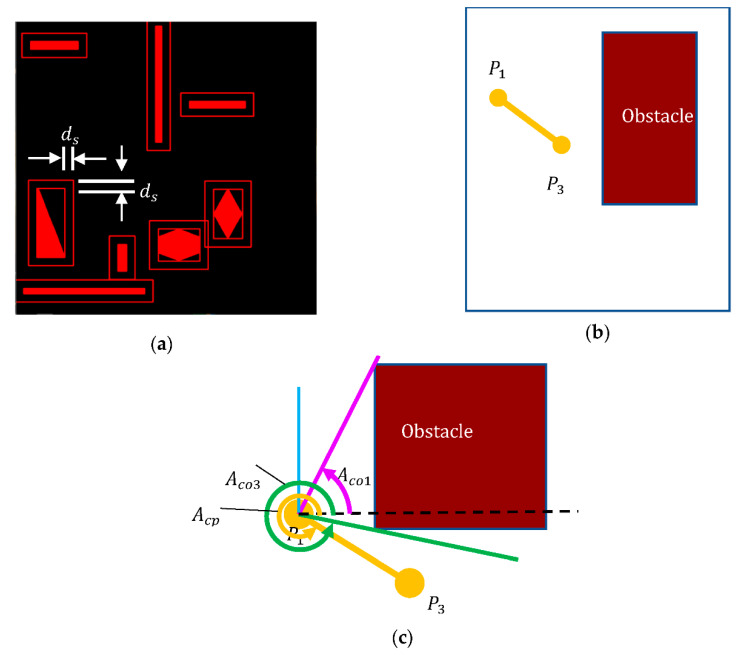
(**a**) The obstacle definition by a rectangle boundary with safety $d_{s}$. Illustration of collision detection when two checkpoints *P_1_* and *P_3_* are (**b**) at the same side or (**c**) at different sides of the obstacle. The latter then requires calculation of the angle $A_{co1}$, $A_{co2}$, and $A_{cp}$ in a collision check.

In path planning, collision detection is necessary to check whether the path between the two checkpoints, say $P_{1}$ and $P_{3}$, is in contact with any obstacle in the space. Polygon modeling for collision detection is often too complicated, time-consuming, and computationally complex, and most choose to simplify the obstacle’s geometric shape for computational efficiency. Consider the obstacle as a rectangle with safety boundary $\;d_{s}$, which is set at half the vehicle’s width for autonomous vehicle applications, as shown in Figure 3a. In a collision check, if both checkpoints and their connection are on the same side of the obstacle, as in Figure 3b, the path after pruning is considered safe. Conversely, if the two checkpoints are on different sides of an obstacle, then one has to check:

(1) $$\{\begin{array}{l} \left(A_{cp}\leq A_{co1}\right)\cup^{\;}\left(A_{co3}\leq A_{cp}\right)\\ A_{co1}\leq A_{cp}\leq A_{co3} \end{array}\;\begin{array}{l} \;if\;P_{1}\;is\;on\;the\;left\;of\;the\;obstacle\\ \;if\;P_{3}\;is\;on\;the\;right\;of\;the\;obstacle \end{array}$$

where $A_{co1}$ and $A_{co3}$ are the angles of the connection between the path points and the corner(s) of the obstacle, and $A_{cp}$ is the angle of the connection between the two checkpoints, as shown in Figure 3c. This collision detection will ensure path safety in the pruning process. For the example of path planning in the obstacle environment shown in Figure 2b, the number of nodes is reduced significantly by 99% after pruning. Such pruning paves the way for efficient path planning, in terms of path length and number of turns, for reaching the destination. With pruning, the unnecessary turns along the path taken by the RRT algorithm will no longer affect the autonomous vehicles.

## 4. Improved RRT Algorithm with Smoothing and Optimization

After the pruning process, a simplified path as shown in Figure 2b was obtained but the path was still not continuously differentiable. There remained several turning points in the path to the destination. This work applied the Bézier curve to generate a continuously differentiable path to the destination. The advantage is its simple implementation and thus comparably low computation cost to guarantee kinematic feasibility while avoiding obstacles. A Bézier curve is often adopted in computer graphics to obtain a continuously differentiable curve tangent to the two lines connecting the adjacent control points. It has also been used in the lane change [33] and path planning [34] of intelligent vehicles. The quadratic Bézier curve (n=2) is written as

(2) $$B\left(t\right)=\overset{n}{\underset{i=0}{\sum }}P_{i+1}b_{i,n}\left(t\right),\;t\in \left[0,\;1\right]$$

where $P_{i+1}$ are the control points of the Bézier curve and the polynomial $b_{i,n}\left(t\right)$ is

(3) $$b_{i,n}\left(t\right)=\left(\begin{array}{c} n\\ i \end{array}\right)t^{i}{\left(1-t\right)}^{n-i},\;i=0,\;1,\;2,\;\ldots ,n$$

**Figure 4:**
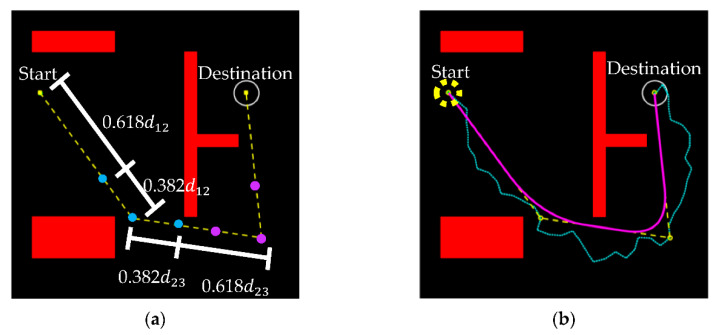
(**a**) Illustration of using two control points for smoothing each turning point in the path by a Bézier curve and (**b**) comparison of the paths predicted by the RRT algorithm (the zigzag) and by the improved RRT algorithm with the pruning and smoothing (the smooth) of this work.

The optimal way to select the control points is to take a turning point, say $P_{2}$, as the center point and follow the path (after pruning) forward and backward a certain distance, at a ratio set at 0.382 (Fibonacci number) from each turning point to the adjacent path points. Two sets of control points for the quadratic Bézier curves are shown in Figure 4a. By this process, a Bézier curve can be obtained at each turning point for a smooth path, as shown in Figure 4b. The calculation in low order of a Bézier curve is simple and the results provide good performance without computational burden. 

**Figure 5:**
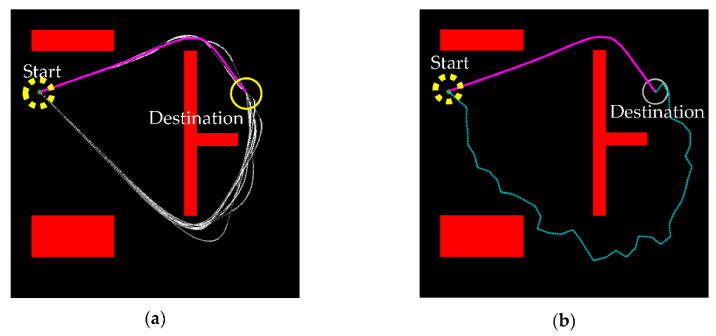
(**a**) The result of the improved RRT algorithm in planning of 10 paths during optimization, and (**b**) among the 10 paths, the one in the shortest path (solid line) is much more efficient than the path taken by the RRT algorithm (dot line) in reaching the destination.

The random nature of the RRT algorithm makes it capable of finding a path to the destination in complex space, but there definitely will be more than one path to the destination. For example, there are many feasible paths, upward or downward, as illustrated in Figure 5a. In terms of path length, the downward path is not as efficient. It is thus important to find a “near”-optimal path for efficient planning. The path planning will be repeated to yield multiple results, which are then compared to find the desired path. To ensure safety, the path points are checked to see whether they are in free space during every planning stage of the improved RRT algorithm. If all path points are in free space, the result of the smoothing process is retained as a candidate path; conversely, the path is abandoned and the planning repeats until a path is found.

Table 1 lists the success rate and average calculation time of the optimization process in one, five, and ten repeated planning steps. The success rate of one-time planning, i.e., no optimization process, is 56%, with 0.09 s of calculation time. The success rate of five-time planning is 74% within 0.42 s, and that of ten-time planning is 96% within 0.90 s of calculation time. The results show that increasing the number of planning steps will increase the chances of finding a better path. The calculation time is acceptable with the advent of high-performance onboard computers. Figure 5a shows the results of 10 repeated planning steps, where the shortest is selected to be the path of the improved RRT algorithm. 

**Figure 6:**
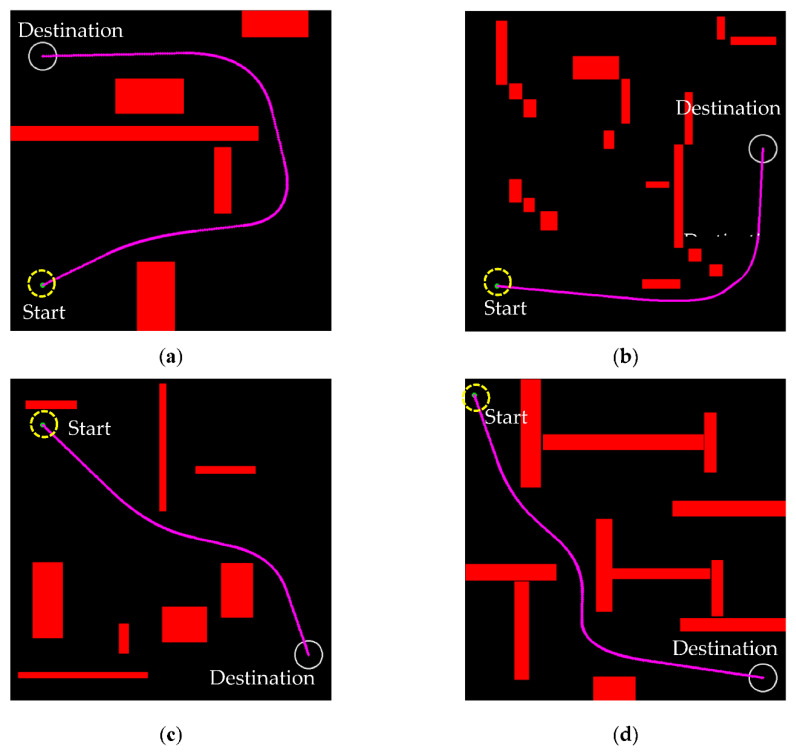
The results of path planning in four different obstacle avoidance environments (**a**–**d**) validate the effectiveness and efficiency of the improved RRT algorithm.

The results show that after the pruning and optimization process, the path length is reduced by around one half, as shown in Figure 5b, and the planning efficiency is significantly improved. It should be noted that the time needed from the start to the destination may be just as important. In this study, the path points were pruned significantly and smoothing was applied to reduce the sharp turns. The path length was considered a good indicator of optimal path planning. Table 2 shows the path length by the RRT algorithm and the improved algorithm with pruning and/or optimization processes. The length was reduced from 1043 to 776, a reduction of around 34% by pruning. The path length was further reduced to 583, around another 33% by optimization. Figure 6 shows the result of the improved RRT algorithm in different obstacle environments. The improved algorithm is an effective path planner for an efficient, continuously differentiable, safe path.

## 5. Experimental Verification by Tracking Control

**Figure 7:**
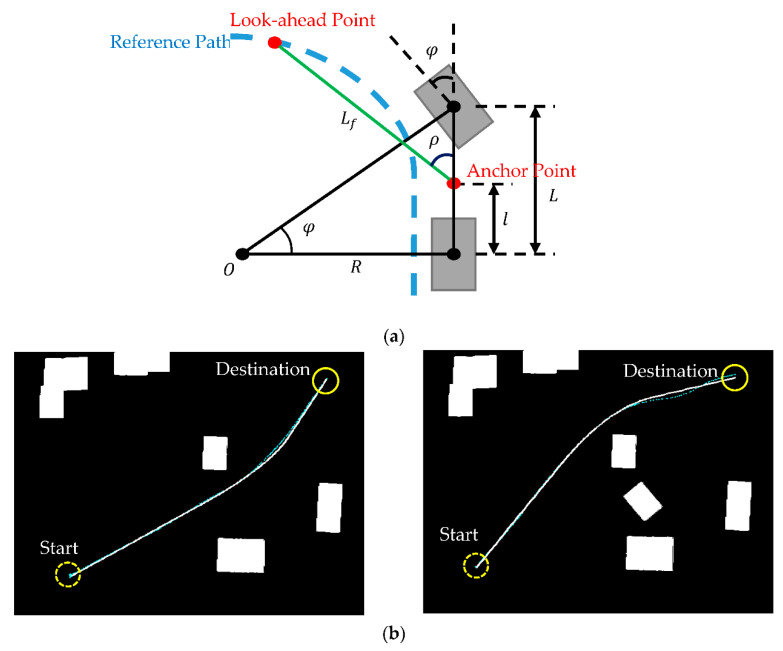
(**a**) The kinematic model of vehicle dynamics with the steering angle φ, the wheelbase L, the distance from the rear wheel axle to the forward anchor point l, the forward drive look-ahead distance $L_{f}$ and the heading of the look-ahead point from the forward anchor point ρ on a path with a radius of curvature *R*. (**b**) Experimental results of the autonomous vehicle tracking the planned path in the obstacle 1 environment (left) and the obstacle 2 environment (right), where the vehicle trajectory of the tracking control (thin line) successfully follows the path predicted by the improved RRT algorithm (solid line) in both obstacle environments.

In order to validate the effectiveness of the improved RRT algorithm in obstacle avoidance, an autonomous vehicle with a pure pursuit controller and a proportional–integral (PI) speed controller was applied to track the planned path. The pure pursuit controller was used to set a look-ahead point at a fixed distance in front of the vehicle from its current position. The geometric relationship between the vehicle and the look-ahead point, as illustrated in Figure 7a, can be defined to obtain the control command of the steering angle $\varphi =\tan^{-1}\left(L/R\right)$, where L is the vehicle wheelbase, $R=\left(L_{f}/2+l\cos\rho \right)/\sin\rho$ is the radius of curvature with respect to the rotation center O of the vehicle, $L_{f}$ is the forward drive look-ahead distance, l is the distance from the rear axle (for rear wheel drive) to the forward anchor point, and ρ is the heading of the look-ahead point (constrained on the planned path) from the forward anchor point with respect to the vehicle heading. Details of the vehicle kinematics model can be found in [35]. A PI controller, $u_{s}=K_{p}\left(V_{cmd}-V_{s}\right)+K_{i}\int_{0}^{t}\left(V_{cmd}-V_{s}\right)d\tau$, is used for speed control, where $u_{s}$ is the speed control command, $K_{p}$ and $K_{i}$ are the proportional and integral gains, respectively, $V_{cmd}$ is the command velocity, and $V_{s}$ is the vehicle speed. The advantage of using the steering controller and speed controller is that they can provide good results with minimal computation load. Advanced controller design can also be implemented on the onboard computer with sufficient computation power. 

**Figure 8:**
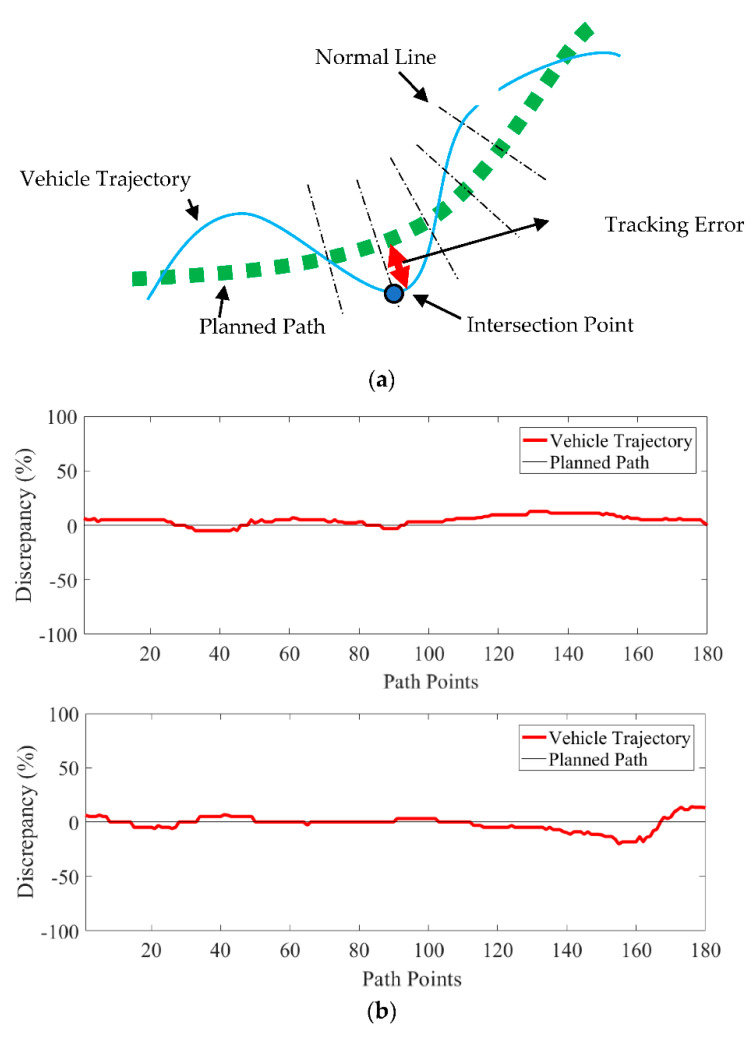
(**a**) Illustration of the trajectory error defined by the distance between the path point and the vehicle’s trajectory point in the experiment, and (**b**) the tracking error in the obstacle 1 environment (above) and the obstacle 2 environment (below).

The experiment was conducted in a static obstacle environment, and the criterion for success was the autonomous vehicle’s capability of traveling along the planned path safely, from the start to the destination, without colliding with obstacles at the speed generating l g acceleration upon turning. For the vehicle wheelbase L= 26 cm, the distance from the rear axle to the forward anchor point l=6 cm, $V_{cmd}=1$ m/s, $L_{f}=\;$50 cm, and the control gains $K_{p}=0.3$ and $K_{i\;}=0.04$, the vehicle followed the planned path of the improved RRT algorithm to the destination on the upper right in Figure 7b. The vehicle trajectory was captured by an observer camera for verifying the improved algorithm. The deviation of the vehicle trajectory from the planned path was defined as the tracking error, as shown in Figure 8a. For the two obstacles in Figure 7b, the tracking errors shown in Figure 8b had an average tracking error of 4.9% and 4.7% of the vehicle width in the two obstacle environments, respectively. The maximum deviation of 12.6% and 20.5%, respectively, was from the vehicle’s initial heading not aligning relative to the planned path, such that the vehicle had to maneuver to align with the path. The results show that the vehicle can track the planned path to the destination without colliding with obstacles. The tracking controller and the speed controller, though classical, were effective. For applications in robotics, some systems may have driving wheels with differential speeds or universal wheels. The vehicle dynamics will definitely have an influence on the controller design. Nevertheless, the path planning by the improved RRT algorithm remains applicable, for the predicted path was continuously differentiable, smooth, and efficient.

**Figure 9:**
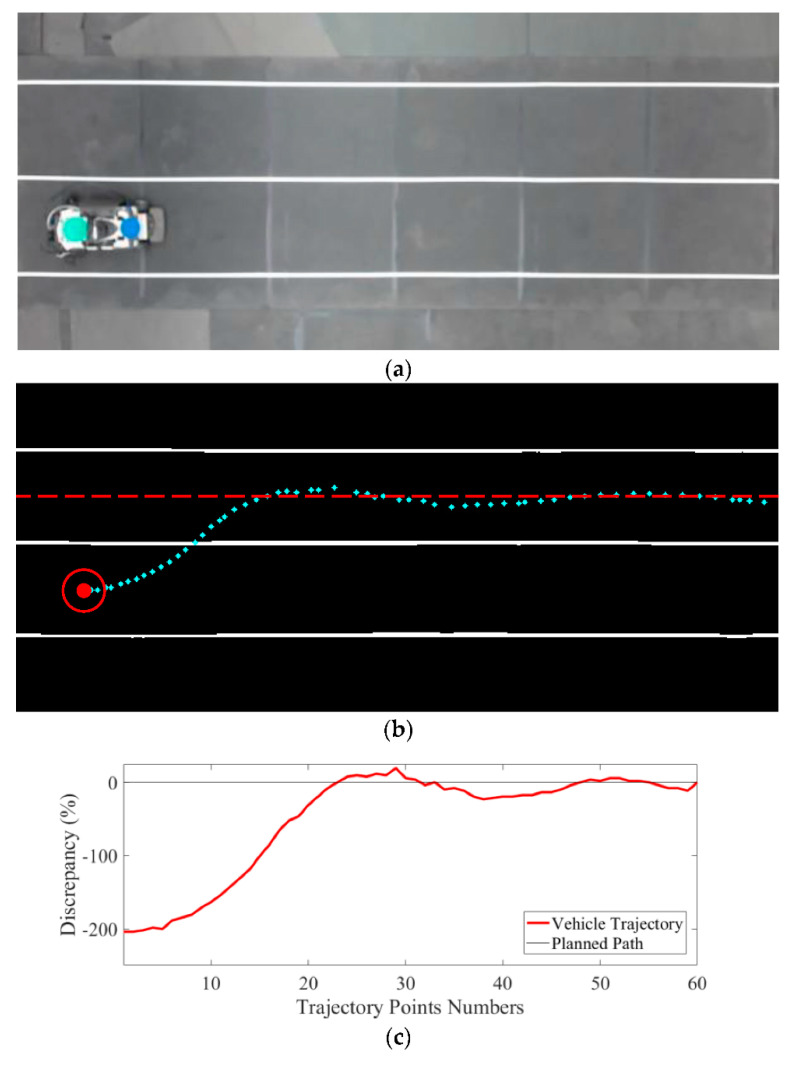
(**a**) Experimental verification of the improved RRT algorithm in the lane change and lane keeping of an autonomous vehicle, (**b**) the vehicle trajectory in lane change and lane keeping, and (**c**) the trajectory discrepancy within 8.3% of vehicle width after the lane change.

Lane change is critical to the development of autonomous vehicles, and it can be modeled as obstacle avoidance. A vehicle parked by the side of the road or a stalled vehicle in the lane ahead require similar responses to obstacle avoidance, and lane change is necessary. Determining the time to execute lane change [36] and following the lane after lane change [37] are also considered obstacle avoidance with regard to autonomous vehicles. With the advent of vehicular social networks, busy traffic spots can be modeled as obstacles. Risk assessment for collision avoidance of nearby obstacles/vehicles will be desirable in future autonomous vehicle development [38]. A decision-making algorithm of risk assessment for collision avoidance was recently proposed for vehicles with different driving style preferences [39]. In this work, the improved RRT algorithm was also verified by the experiment that combined lane change and lane keeping. For an autonomous vehicle on a two-lane road, as in Figure 9a, if there is an obstacle in front of the current lane, the vehicle needs to change lanes and continue lane keeping afterward. The experimental result of lane change is shown in Figure 9b. Starting from the lower left (say, outer lane), the vehicle successfully tracked the upper lane (say, inner lane) after the lane change. Figure 9c shows the discrepancy in the combined lane change and lane keeping to the upper lane. The maximum discrepancy of over 200% was in the initiation stage of lane change because the vehicle’s initial position was far from the center of the upper lane. The discrepancy in lane keeping remained within 21.4% after the lane change, with an average of 8.3% of the vehicle width. The result also validated that the improved RRT algorithm was efficient in path planning, and it was also effective in combined lane change and lane keeping. It should be noted that the proposed algorithm may be limited by the time needed to re-plan a safe path should an obstacle “suddenly” appear ahead. Care then has to be taken in risk assessment [38,39].

## 6. Conclusions

An improved RRT algorithm was developed for the path planning of autonomous vehicles in static obstacle avoidance. The algorithm integrates (a) the pruning process with geometric collision detection to reach an efficient and collision-free safe path, (b) the smoothing process by the quadratic Bézier curve to obtain a continuously differentiable path for vehicle implementation, and (c) the optimization process to select the relatively superior path in terms of path length as the final path. Simulation results show that the improved RRT algorithm can plan a collision-free, safe path from the start to the destination in multiple obstacle environments. It has been shown that the pruning process would substantially reduce the number of turning points in the path by 99% compared with the RRT algorithm. The smoothing process by the quadratic Bézier curves with the control points set at Fibonacci number would further avoid the “sharp” turns along the path distance. 

For the path planning example in Figure 5 with the search repeated 10 times, the success rate in the optimization process of finding a desired path is 96%. The path length is reduced by 34% after pruning and by another 33% after optimization. In the experimental verification on autonomous vehicles, a pure pursuit controller and a PI controller were applied to track the desired, planned path by the improved RRT algorithm. For a vehicle speed of 1 m/s within l g acceleration upon turning, it was experimentally validated that the vehicle could track the planned path to reach the destination safely. The average tracking deviation in the two environments was 4.9% and 4.7%, respectively, of the vehicle width. Note that the deviation can be further reduced by advanced controller(s), yet for path planning through a maze of narrow corridors, the constraint of vehicle dynamics of minimum turning radius should be investigated.

Lane change is considered similar to obstacle avoidance in autonomous vehicles. The combined lane change and lane keeping was also verified by the experiment. After the lane change, the discrepancies in lane keeping remained within 8.3% of the vehicle width. The results show that the improved RRT algorithm can also be applied to combined lane change and lane keeping. It should be noted that the proposed algorithm may be limited by the time needed to re-plan a safe path should an obstacle “suddenly” appear ahead. With the advent of vehicular social networks, busy traffic spots can be modeled as obstacles. Risk assessment for collision avoidance of nearby obstacles/vehicles [39] will be desirable in future autonomous vehicle development. 

## Figures and Tables

**Table 1:** The success rate and average calculation time for 1, 5, and 10 planning steps of the improved RRT algorithm in obtaining a “near”-optimal path.

Number of Repetitions	Success Rate (%)	Average Calculation Time (ms)
1	56	91
5	74	420
10	96	900

**Table 2:** The average path length by the RRT algorithm, the improved algorithm with pruning, and the improved algorithm with pruning and optimization.

RRT Algorithm	With Pruning	With Pruning and Optimization
1043	776	583

## Data Availability

The data presented in this study are available upon request.

## References

[1] World Health Organization (2018). Global Status Report on Road Safety 2018.

[2] Rahim A., Kong X., Xia F., Ning Z., Ullah N., Wanga J., Das S.K. (2018). Vehicular Social Networks: A Survey. Sci. Direct Pervasive Mob. Comput..

[3] González D., Pérez J., Milanés V., Nashashibi F. (2016). A Review of Motion Planning Techniques for Automated Vehicles. IEEE Trans. Intell. Transp. Syst..

[4] Rosique F., Navarro P.J., Fernandez C., Padilla A. (2019). A Systematic Review of Perception System and Simulators for Autonomous Vehicles Research. Sensors.

[5] Claussmann L., Revilloud M., Gruyer D., Glaser S. (2020). A Review of Motion Planning for Highway Autonomous Driving. IEEE Trans. Intell. Transp. Syst..

[6] Shoaib A., Farzeen M., Muqeem S.A., Jeon M. (2020). System, Design and Experimental Validation of Autonomous Vehicle in an Unconstrained Environment. Sensors.

[7] de Winter A., Baldi S. (2018). Real-Life Implementation of a GPS-Based Path-Following System for an Autonomous Vehicle. Sensors.

[8] Wen W.S., Hsu L.T., Zhang G.H. (2018). Performance Analysis of NDT-based Graph SLAM for Autonomous Vehicle in Diverse Typical Driving Scenarios of Hong Kong. Sensors.

[9] Yang X., Xiong L., Leng B., Zeng D.Q., Zhuo G.R. (2020). A Global Path Planner for Safe Navigation of Autonomous Vehicles in Uncertain Environments. Sensors.

[10] Fayyad J., Jaradat M.A., Gruyer D., Najjaran H. (2020). Deep Learning Sensor Fusion for Autonomous Vehicle Perception and Localization: A Review. Sensors.

[11] Haris M., Hou J. (2020). Obstacle Detection and Safely Navigate the Autonomous Vehicle from Unexpected Obstacles on the Driving Lane. Sensors.

[12] Receveur J., Victor S., Melchior P. (2020). Autonomous Car Decision Making and Trajectory Tracking Based on Genetic Algorithms and Fractional Potential Fields. Intell. Serv. Robot..

[13] Wang N., Xu H.W., Li C.Z., Yin J.C. Hierarchical Path Planning of Unmanned Surface Vehicles: A Fuzzy Artificial Potential Field Approach. Int. J. Fuzzy Syst..

[14] Wang X.H., Liang Y., Liu S., Xu L.F. (2019). Bearing-Only Obstacle Avoidance Based on Unknown Input Observer and Angle-Dependent Artificial Potential Field. Sensors.

[15] Lu B., Li G.F., Yu H.L., Wang H., Guo J.Q., Cao D.P., He H.W. (2020). Adaptive Potential Field-Based Path Planning for Complex Autonomous Driving Scenarios. IEEE Access.

[16] Ayawli B.K., Chellali R., Appiah A.Y., Kyeremeh F. (2018). An Overview of Nature-Inspired, Conventional, and Hybrid Methods of Autonomous Vehicle Path Planning. J. Adv. Transp..

[17] Wang N., Xu H.W. (2020). Dynamics-Constrained Global-Local Hybrid Path Planning of an Autonomous Surface Vehicle. IEEE Trans. Veh. Technol..

[18] Lu B., He H.W., Yu H.L., Wang H., Li G.F., Shi M., Cao D.P. (2020). Hybrid Path Planning Combining Potential Field with Sigmoid Curve for Autonomous Driving. Sensors.

[19] Nielsen L.D., Sung I., Nielsen P. (2019). Convex Decomposition for a Coverage Path Planning for Autonomous Vehicles: Interior Extension of Edges. Sensors.

[20] Martinez R., Jimenez F. (2019). Implementation of a Potential Field-Based Decision-Making Algorithm on Autonomous Vehicles for Driving in Complex Environments. Sensors.

[21] Lavelle S.M. (2001). Rapidly-Exploring Random Trees: Progress and Prospects. in Algorithmic Comput. Robot..

[22] LaValle S.M., Kuffner J.J. (2001). Randomized Kinodynamic Planning. Int. J. Robot. Res..

[23] Zhang H., Wang Y., Zheng J., Yu J. (2018). Path Planning of Industrial Robot Based on Improved RRT Algorithm in Complex Environments. IEEE Access.

[24] Sakcak B., Bascetta L., Ferretti G., Prandini M. (2019). Sampling-Based Optimal Kinodynamic Planning with Motion Primitives. Auton. Robot..

[25] Ravankar A., Ravankar A.A., Kobayashi Y., Hoshino Y., Peng C.-C. (2018). Path Smoothing Techniques in Robot Navigation: State-of-the-Art, Current and Future Challenges. Sensors.

[26] Noreen I., Khan A., Asghar K., Habib Z. (2019). A Path-Planning Performance Comparison of RRT*-AB with MEA* in a 2-Dimensional Environment. Symmetry.

[27] Wei K., Ren B. (2018). A Method on Dynamic Path Planning for Robotic Manipulator Autonomous Obstacle Avoidance Based on an Improved RRT Algorithm. Sensors.

[28] Xu J.J., Park K.S. Moving Obstacle Avoidance for Cable-Driven Parallel Robots Using Improved RRT. Microsyst. Technol. Micro Nanosyst. Inf. Storage Process. Syst..

[29] Kang J.G., Lim D.W., Choi Y.S., Jang W.J., Jung J.W. (2021). Improved RRT-Connect Algorithm Based on Triangular Inequality for Robot Path Planning. Sensors.

[30] Yuan C.R., Liu G.F., Zhang W.Q., Pan X.L. (2020). An Efficient RRT Cache Method in Dynamic Environments for Path Planning. Robot. Auton. Syst..

[31] Blanco J.L., Bellone M., Gimenez-Fernandez A. (2015). TP-Space RRT-Kinematic Path Planning of Non-Holonomic Any-Shape Vehicles. Int. J. Adv. Robot. Syst..

[32] Mischinger M., Rudigier M., Wimmer P., Kerschbaumer A. (2019). Towards Comfort-Optimal Trajectory Planning and Control. Veh. Syst. Dyn..

[33] Chen L., Qin D., Xu X., Cai Y., Xie J. (2019). A Path and Velocity Planning Method for Lane Changing Collision Avoidance of Intelligent Vehicle Based on Cubic 3-D Bezier Curve. Adv. Eng. Softw..

[34] Li H., Luo Y., Wu J. (2019). Collision-Free Path Planning for Intelligent Vehicles Based on Bézier Curve. IEEE Access.

[35] Kuo C.Y., Lu Y.R., Yang S.M. (2019). On the Image Sensor Processing for Lane Detection and Control in Vehicle Lane Keeping Systems. Sensors.

[36] Wang C., Sun Q.Y., Li Z., Zhang H.J. (2020). Human-Like Lane Change Decision Model for Autonomous Vehicles that Considers the Risk Perception of Drivers in Mixed Traffic. Sensors.

[37] Lin F., Wang K.Z., Zhao Y.Q., Wang S.B. (2020). Integrated Avoid Collision Control of Autonomous Vehicle Based on Trajectory Re-Planning and V2V Information Interaction. Sensors.

[38] Zhang L.J., Xiao W., Zhang Z., Meng D.J. (2020). Surrounding Vehicles Motion Prediction for Risk Assessment and Motion Planning of Autonomous Vehicle in Highway Scenarios. IEEE Access.

[39] Li G.F., Yang Y.F., Zhang T.R., Qu X.D., Cao D.P., Cheng B., Li K.Q. (2021). Risk Assessment Based Collision Avoidance Decision-Making for Autonomous Vehicles in Multi-Scenarios. Transp. Res. Part C Emerg. Technol..

